# Efficacy of Second-Line Biological Therapies in Moderate to Severe Ulcerative Colitis Patients with Prior Failure of Anti-Tumor Necrosis Factor Therapy: A Multi-Center Study

**DOI:** 10.3390/jpm14101066

**Published:** 2024-10-18

**Authors:** Ji-Eun Na, Yong-Eun Park, Jong-Ha Park, Tae-Oh Kim, Jong-Yoon Lee, Jong-Hoon Lee, Su-Bum Park, Seung-Bum Lee, Seung-Min Hong

**Affiliations:** 1 Department of Internal Medicine, Inje University, Haeundae Paik Hospital, Busan 48108, Republic of Korea; h00609@paik.ac.kr (J.-E.N.); ready200@paik.ac.kr (Y.-E.P.); h00095@paik.ac.kr (J.-H.P.); 2 Department of Internal Medicine, Dong-A University, College of Medicine, Busan 49201, Republic of Korea; ljyhateo@dau.ac.kr (J.-Y.L.); jh2002@dau.ac.kr (J.-H.L.); 3 Department of Internal Medicine, Pusan National University Yangsan Hospital, Yangsan 50612, Republic of Korea; psubumi@pusan.ac.kr; 4 Department of Gastroenterology, Ulsan University Hospital, University of Ulsan College of Medicine, Ulsan 44033, Republic of Korea; sblee@uuh.ulsan.kr; 5 Department of Internal Medicine, Pusan National University School of Medicine and Biomedical Research Institute, Pusan National University Hospital, Busan 49241, Republic of Korea; lucky77i@pusan.ac.kr

**Keywords:** moderate to severe ulcerative colitis, prior exposure to tumor necrosis factor antagonists, efficacy and safety between second-line biological therapies

## Abstract

Background: Few studies have compared the efficacy and safety of second-line biological therapies in ulcerative colitis (UC) patients with prior exposure to anti-tumor necrosis factor (TNF) therapy. We aim to compare the efficacy and safety between ustekinumab, vedolizumab, and tofacitinib, a current option as second-line biological therapy with different mechanisms in those patients. Methods: This retrospective multi-center study was conducted across five institutions from 2011 to 2022. We enrolled patients with moderate to severe UC who failed anti-TNF therapy and subsequently received ustekinumab, vedolizumab, or tofacitinib as second-line biological therapy. The outcomes were analyzed for clinical response/remission and endoscopic improvement/remission rates after induction therapy, drug persistency, and adverse events. Results: A total of 70 UC patients were included and grouped into ustekinumab (11 patients), vedolizumab (40 patients), and tofacitinib (19 patients) treatments. The clinical response/remission rates after induction therapy were similar between ustekinumab (90.9/81.8%), vedolizumab (92.5/65.0%), and tofacitinib (94.7/73.7%). There were no significant differences in the endoscopic improvement/remission rates between the three groups: 90.9/18.2% for ustekinumab, 72.5/12.5% for vedolizumab, and 84.2/26.3% for tofacitinib. Drug persistence was similar across the three agents (*p* = 0.130). Three patients of the tofacitinib group experienced adverse events (herpes zoster and hypertriglyceridemia). Conclusions: Based on real-world data, second-line biological therapy with ustekinumab, vedolizumab, and tofacitinib showed comparable efficacy in patients with moderate to severe UC with prior exposure to anti-TNF therapy.

## 1. Introduction

Patients with moderate to severe ulcerative colitis (UC) have been reported to experience a primary non-response (PNR) rate of 13% to 49% to anti-tumor necrosis factor (anti-TNF) therapy [1,2,3,4,5], with an annual loss of response (LOR) reported to range from 10.1% to 13.6% [6]. Subtherapeutic drug concentrations and developing antidrug antibodies are among the causes of PNR or LOR. In such scenarios, therapeutic drug monitoring can guide the addition of immunomodulators for combination therapy or switching treatments within or out of class [7,8]. However, combination therapy may be associated with an increased risk of opportunistic infections or malignancies [9]. For UC patients with prior exposure to anti-TNF therapy, new biologics and small-molecule drugs targeting different pathways, such as an α4β7 integrin antagonist, a human monoclonal antibody against interleukin 12 (IL-12) and IL-23, and a Janus kinase (JAK) inhibitor, are currently available as options for second-line biological therapy.

Despite the diversity in treatment options, the selection algorithm has become more complex, and a lack of comparative data has led to the absence of a consensus on the optimal second-line biological therapy for UC patients previously exposed to anti-TNF therapy. The American Gastroenterological Association guideline suggests using ustekinumab or tofacitinib over vedolizumab or adalimumab in UC patients who have failed infliximab therapy [10]. This recommendation was supported by a recent large-scale retrospective study [11]. However, the strength of this recommendation is limited, as it is based on a single network meta-analysis (NMA) [12]. NMAs combine data from multiple studies with varying designs, which can introduce heterogeneity and complicate outcome comparisons [13]. Other guidelines present ustekinumab, vedolizumab, and tofacitinib as equivalent options [14,15,16]. Recent literature supports this view, showing no significant difference in clinical remission rates after induction therapy between vedolizumab and tofacitinib in UC patients with prior anti-TNF treatment failure [17].

Owing to a lack of relevant data, an unmet clinical question remains regarding whether an optimal treatment sequence exists when considering comprehensive clinical factors for patients with moderate to severe UC with prior anti-TNF treatment failure. To address this question, we used real-world data to compare the efficacy and safety of ustekinumab, vedolizumab, and tofacitinib in patients with moderate to severe UC with prior anti-TNF exposure.

## 2. Methods

### 2.1. Patients

This is a multi-center retrospective cohort study from five general and tertiary hospitals. Adult patients with moderate to severe UC who had failed anti-TNF therapy were eligible from January 2011 to December 2022. Anti-TNF failure included PNR, LOR, and adverse events. Only patients treated with second-line biological therapies (ustekinumab, vedolizumab, or tofacitinib) who were followed for at least six months were enrolled; all were naïve to these agents. The Institutional Review Board of Haeundae Paik Hospital approved our protocol (file number 2022-07-014-001). The patients’ informed consent requirement was waived because only de-identified data were collected.

### 2.2. Outcomes and Assessment

The primary outcome was to compare clinical response/remission and endoscopic response/remission rates after induction therapy, drug persistency, and adverse events during second-line biological therapy (ustekinumab, vedolizumab, or tofacitinib). The secondary outcome was to identify factors associated with the discontinuation of second-line biological therapy.

The index date was the start date of second-line biological therapy. The schedule of induction and maintenance therapy and response assessment after induction therapy was as follows: (1) intravenous infusion of ustekinumab at week 0 according to body weight (260 mg for ≤55 kg, 390 mg from >55 kg to ≤85 kg, or 520 mg for >85 kg); subcutaneous ustekinumab 90 mg at week 8, and maintained every 12 weeks, which could be shortened to 8 weeks depending on the clinician’s decision. Response after induction therapy was evaluated before the third dose around weeks 16 to 20. (2) Intravenous infusion of 300 mg of vedolizumab at weeks 0, 2, and 8. And it was maintained every 8 weeks, which could be shortened to 4 weeks. After the third dose, response after induction therapy was assessed around week 14. (3) An amount of 10 mg of tofacitinib twice daily orally for 8 weeks and maintained at 5 mg or 10 mg twice daily; response after induction therapy was checked around week 16.

In South Korea, the Mayo score should be evaluated before and after the induction of biological therapy in patients with moderate to severe UC due to insurance policies. The Mayo score items consist of stool frequency, rectal bleeding, findings at proctosigmoidoscopy, and physician’s global assessment, which are scored from 0 to 3, respectively [18]. Clinical response after induction therapy was defined as a decrease of 50% or more in the sum of the bowel frequency and rectal bleeding score [19]. Clinical remission was set as a partial Mayo of less than 3, with no individual item exceeding 1 point [19]. Endoscopic improvement and remission were defined as a Mayo endoscopic subscore (MES) of 0 or 1 and an MES of 0, respectively. The duration of drug persistency was defined as the period from the index date to the last dose, irrespective of whether the patient experienced discontinuation. Reasons for drug discontinuation were categorized as PNR, LOR, or adverse events. PNR was defined as a failure to achieve a clinical response during response assessment after induction therapy. LOR was described as an initial achievement of clinical response or remission after induction, followed by increased stool frequency or worsening rectal bleeding symptoms during maintenance. This led to a Mayo score of 6 or higher, indicating moderate to severe disease activity [20,21]. Enrolled patients were followed until their last outpatient visit and monitored for adverse events.

### 2.3. Covariates

The following variables were collected retrospectively based on medical records at each institution: age at diagnosis, age at the start of second-line biological therapy, disease duration, gender, the extent of disease (classified according to the Montreal classification, where left-sided UC was defined as a disease limited to the distal colon not extending beyond the splenic flexure and extensive UC as a disease extending proximal to the splenic flexure [22]), number of prior anti-TNF therapies, duration of anti-TNF therapy, the reason for discontinuation of anti-TNF therapy (PNR, LOR, or adverse events), disease activity index (Mayo score from 6 to 10/from 11 to 12), concomitant medication [5-aminosalicylic acid (5-ASA), topical 5-ASA, steroid, or immunomodulators], laboratory data [hemoglobin, C-reactive protein (CRP), and albumin], and follow-up duration.

### 2.4. Statistical Analysis

Continuous variables were presented as mean ± standard deviation and compared using a one-way analysis of variance or the Kruskal–Wallis test. Categorical variables were expressed as *n* (%) and compared using the chi-squared or Fisher’s exact test. To compare the efficacy of second-line biological therapy, Fisher’s exact test, a chi-squared test, and the Bonferroni method were used. Drug persistency was analyzed using the Kaplan–Meier curve with a log-rank test. Cox regression analysis was used to identify factors associated with discontinuing second-line biologics. Only elements with a *p* value less than 0.1 in univariable analysis were included in multivariable analysis, and a *p* value < 0.05 was considered statistically significant. Statistical analysis was performed using SPSS Statistics 25.0 and R-4.2.2.

## 3. Result

### 3.1. Baseline Characteristics

A total of seventy patients with moderate to severe UC who had prior exposure to anti-TNF therapy were identified and grouped into three treatment cohorts: ustekinumab (N = 11), vedolizumab (N = 40), and tofacitinib (N = 19). The baseline characteristics of the patients at the initiation of second-line biological therapy are summarized in Table 1. The age at diagnosis was similar across all three groups, ranging from 36.0 ± 15.9 years in the ustekinumab group to 37.8 ± 17.2 years in the vedolizumab group, with the tofacitinib group having an average of 36.5 ± 14.5 years. There was no statistically significant difference in the age of diagnosis between the three treatment groups. Similarly, the age at the initiation of second-line biologic therapy was comparable across the groups, with no significant difference observed. The ustekinumab group initiated second-line therapy at an average age of 43.8 ± 21.5 years, while the vedolizumab and tofacitinib groups started at 43.6 ± 16.8 and 41.1 ± 13.5 years, respectively. There was no significant difference in sex distribution among the groups, although a higher proportion of males was observed in the vedolizumab (67.5%) and tofacitinib (73.7%) groups compared to the ustekinumab group (45.5%). The extent of disease was classified into left-sided colitis and extensive colitis. The proportion of patients with extensive colitis was lower in the ustekinumab group (36.4%) compared to the vedolizumab (50.0%) and tofacitinib (47.4%) groups, though this difference was not statistically significant. Most patients had been exposed to only one anti-TNF agent, and the percentage of patients exposed to two or more anti-TNF therapies was less than 10% in all groups. The duration of anti-TNF therapy was longest in the ustekinumab group (45.3 ± 40.4 months) compared to the vedolizumab (19.5 ± 22.3 months) and tofacitinib (11.7 ± 10.8 months) groups, but no significant differences were found between the groups. Among the reasons for discontinuing anti-TNF therapy, loss of response (LOR) accounted for the highest proportion in all groups: 63.6% in the ustekinumab group, 72.5% in the vedolizumab group, and 73.7% in the tofacitinib group. The disease activity index (Mayo score) indicated that most patients had moderate disease activity, while the proportion of patients with severe disease was higher in the ustekinumab group (27.3%) compared to the vedolizumab (12.5%) and tofacitinib (15.8%) groups, though the difference was not statistically significant. As expected, oral 5-ASA was the most commonly used concomitant medication. The proportion of patients using topical 5-ASA was higher in the tofacitinib group (36.8%) compared to the ustekinumab (18.2%) and vedolizumab (10.0%) groups. The use of steroids was highest in the ustekinumab group (63.6%) compared to the vedolizumab (42.5%) and tofacitinib (42.1%) groups, but no statistically significant differences were observed. Immunomodulator use was most prevalent in the vedolizumab group (40.0%) compared to the other second-line biologic therapies. Hemoglobin and albumin levels were similar across all groups, with no significant differences noted. The CRP levels were elevated in all groups, with an average of 0.9 ± 1.4 in the ustekinumab group, 1.4 ± 1.9 in the vedolizumab group, and 0.9 ± 1.0 in the tofacitinib group. The follow-up duration was shorter in the ustekinumab group (mean 16.1 ± 4.9 months) compared to the vedolizumab (mean 34.0 ± 14.4 months) and tofacitinib (mean 31.3 ± 7.2 months) groups.

### 3.2. Outcomes After Induction Therapy

**Figure 1:**
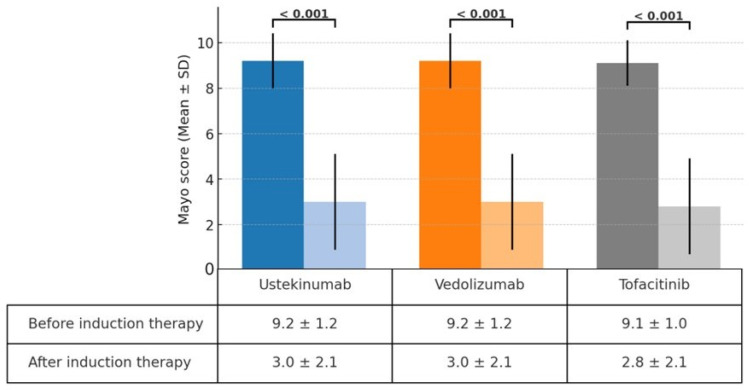
Change in Mayo score before and after induction therapy with ustekinumab, vedolizumab, and tofacitinib.

**Figure 2:**
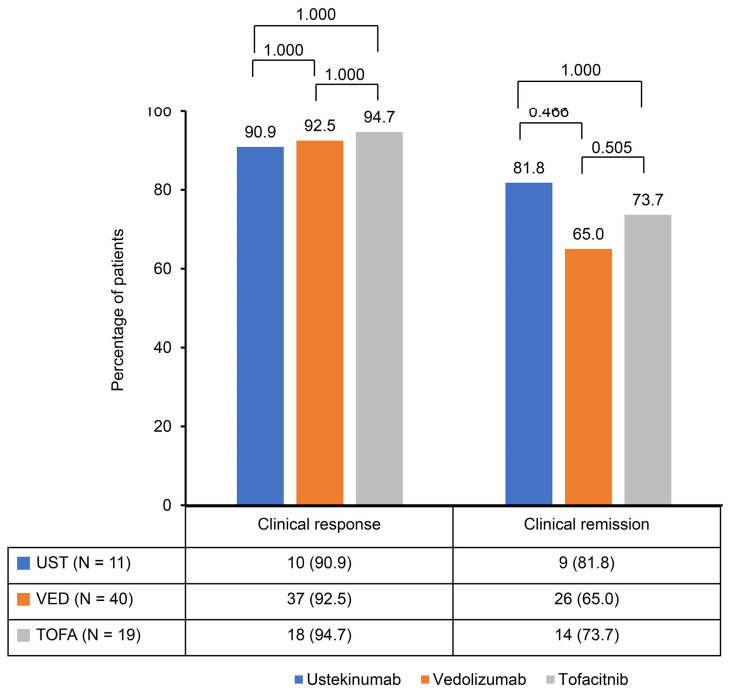
Clinical response and remission rates after induction of second-line biological therapy.

**Figure 3:**
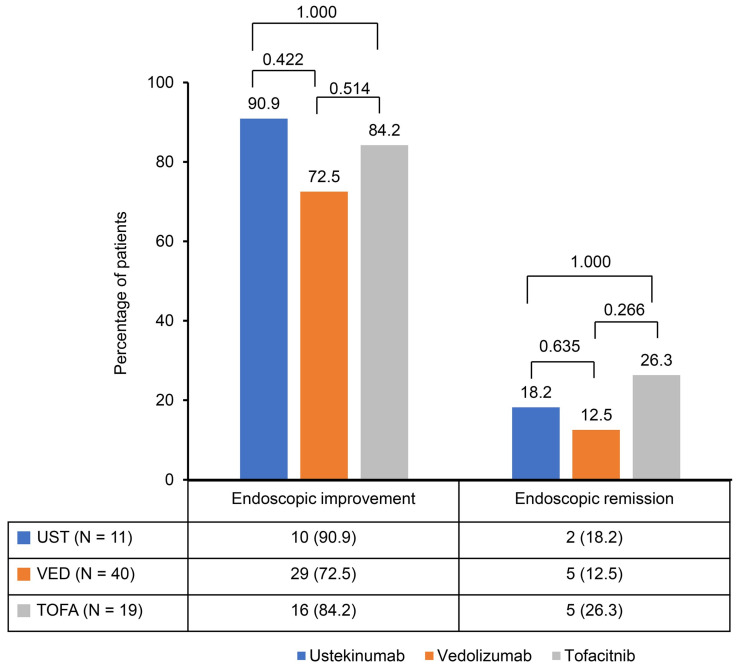
Endoscopic improvement and remission rates after induction of second-line biological therapy.

A significant reduction in the Mayo score was observed in the ustekinumab, vedolizumab, and tofacitinib groups after induction therapy. The Mayo scores decreased from 9.2 ± 1.2 to 3.0 ± 2.1 in the ustekinumab and vedolizumab groups and from 9.1 ± 1.0 to 2.8 ± 2.1 in the tofacitinib group (Figure 1). The clinical response rates following induction therapy were similarly high across all groups, with 90.9% of patients responding in the ustekinumab group, 92.5% in the vedolizumab group, and 94.7% in the tofacitinib group, showing no statistically significant differences between the groups (Figure 2). Although ustekinumab demonstrated a high clinical remission rate of 81.1%, there was no significant difference compared to the rate of 65.0% in the vedolizumab group and 73.7% in the tofacitinib group. Endoscopic remission rates were low: 18.2% for ustekinumab, 12.5% for vedolizumab, and 26.3% for tofacitinib. The endoscopic improvement rates after induction therapy were also high, with 90.9% of patients in the ustekinumab group showing improvement. However, no statistically significant differences were observed when compared to the vedolizumab group (72.5%) and the tofacitinib group (84.2%) (Figure 3). In contrast, endoscopic remission rates were generally low across all groups, with 18.2% in the ustekinumab group, 12.5% in the vedolizumab group, and 26.3% in the tofacitinib group.

### 3.3. Drug Persistency, Safety, and Associating Factors

During the follow-up period, 0 patients (0.0%) with ustekinumab, 14 patients (35.0%) with vedolizumab, and 4 patients (21.1%) with tofacitinib experienced discontinuation (Table 2).

**Figure 4:**
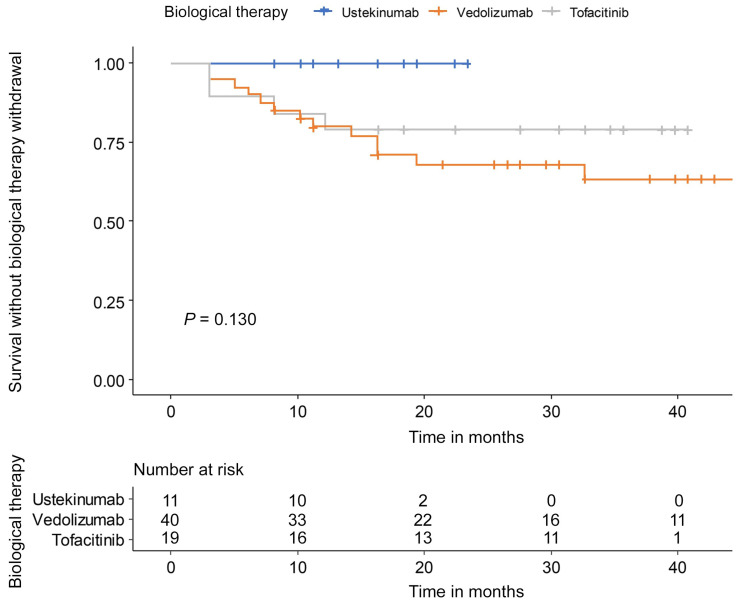
Drug persistency during follow-up of maintenance therapy.

The withdrawal reason was PNR in 2 patients and LOR in 12 patients among the 14 patients of vedolizumab. Four patients of the tofacitinib group stopped because of PNR in one patient, LOR in two patients, and adverse events in one patient. In the tofacitinib group, two patients experienced adverse events but did not discontinue therapy. There was no significant difference in drug persistency between ustekinumab, vedolizumab, and tofacitinib (*p* = 0.130) (Figure 4). Clinical response (HR: 0.09, 95% CI: 0.03–0.31) and endoscopic improvement (HR: 0.15, 95% CI: 0.06–0.41) were associated with a low risk of second-line biological therapy discontinuation (Table 3).

## 4. Discussion

This study observed comparable efficacy among second-line biological therapies—ustekinumab, vedolizumab, and tofacitinib—in patients with moderate to severe UC who had previously failed anti-TNF therapy. Significant reductions in Mayo scores were seen across all groups following induction therapy, with decreases from 9.2 ± 1.2 to 3.0 ± 2.1 in the ustekinumab and vedolizumab groups and from 9.1 ± 1.0 to 2.8 ± 2.1 in the tofacitinib group. This reduction reflects the effectiveness of these therapies in inducing clinical improvement. Clinical response rates were similarly high, with 90.9% of patients responding to ustekinumab, 92.5% to vedolizumab, and 94.7% to tofacitinib. Ustekinumab exhibited a clinical remission rate of 81.1%, which, although higher, was not significantly different from the rates observed in the vedolizumab (65.0%) and tofacitinib (73.7%) groups. Endoscopic improvement rates were also favorable across all therapies, with 90.9% in the ustekinumab group, 72.5% in the vedolizumab group, and 84.2% in the tofacitinib group. However, endoscopic remission rates were generally low, ranging from 12.5% to 26.3% across all groups.

Drug discontinuation rates during follow-up were 0% in the ustekinumab group, 35% in the vedolizumab group, and 21.1% in the tofacitinib group, with no statistically significant differences between the therapies. These findings should be interpreted cautiously, as ustekinumab was introduced as a second-line therapy more recently (in 2021), resulting in a shorter follow-up period compared to vedolizumab and tofacitinib. Additional long-term data are necessary to further evaluate drug persistence. Regarding safety, no adverse events were reported in the ustekinumab and vedolizumab groups. In contrast, three adverse events (herpes zoster and hypertriglyceridemia) occurred in the tofacitinib group, leading to discontinuation in one patient. No major adverse cardiovascular events or venous thromboembolism were observed in the tofacitinib group, consistent with previous post-marketing surveillance conducted in South Korea [23,24]. Achieving clinical response (HR: 0.09, 95% CI: 0.03–0.31) or endoscopic improvement (HR: 0.15, 95% CI: 0.06–0.41) appeared to be associated with a lower risk of drug discontinuation. These results suggest that early improvements in clinical and endoscopic outcomes may contribute to long-term treatment success, although prospective studies are needed to validate these associations. Overall, all three therapies showed promising outcomes, but longer-term studies are required to assess drug durability and mucosal healing in UC patients.

We first compared the efficacy and drug persistency among ustekinumab, vedolizumab, and tofacitinib, currently available for treating moderate to severe UC patients with prior failure of anti-TNF therapy. Real-world data were collected from five institutions, and clinically important indicators, including clinical response/remission, endoscopic improvement/remission, and drug persistency, were used as outcome measures. Although it was not a prospective study, all covariates related to clinical outcomes were collected without missing, as the timing and method for assessing disease activity before and after biologics induction therapy were generally established based on insurance guidance.

A previous study in UC patients who had failed anti-TNF therapy found no significant difference in clinical remission rates after induction therapy between vedolizumab (56.8%) and tofacitinib (46.2%), similar to our results [17]. Another large-scale study reported that, in UC patients previously exposed to TNF inhibitors, vedolizumab was associated with a higher risk of intravenous steroid use or colectomy within two years compared to ustekinumab. However, this study had limitations, such as potential selection bias and including events that may not have been directly related to UC exacerbation [11]. Although the study population was not constricted to UC patients who had failed anti-TNF therapy, another cohort, which included 50% of patients with prior failure of anti-TNF therapy, showed a clinical response rate of 77.6% for ustekinumab at week 16 [25]. For UC patients who mostly (85–98%) experienced anti-TNF therapy, the clinical response/remission rates of vedolizumab at week 14 were 43–57% and 28–39%, respectively [26,27]. In a cohort where half of the UC patients had a suboptimal response to anti-TNF therapy, tofacitinib demonstrated clinical remission and mucosal healing (MES 0 or 1) rates of 18.5% and 31.3%, respectively, at week 8 [28]. Our results demonstrated relatively high response rates compared to the previous literature. The timing of response assessment in our study, conducted between weeks 14 and 20 after induction therapy, may have contributed to these favorable outcomes. Additionally, the concurrent use of steroids in 42% to 64% of patients at the initiation of second-line biologics could have influenced the differences in response rates.

A meta-analysis of second-line biologics for UC patients with prior failure of anti-TNF therapy reported superior efficacy of ustekinumab or tofacitinib rather than vedolizumab [12,29]. However, generalization is limited because the number of studies included in the meta-analysis is small, and the response evaluation was earlier than actual practice. In UC patients with prior failure of anti-TNF therapy, the clinical response/remission rates with vedolizumab therapy were increased at week 14 compared to week 6 [26]. Previously, vedolizumab as a second-line biologic exerted an endoscopic improvement of 50% at week 14 [30], and the clinical response and mucosal healing rate at week 52 was 44.6% [31]. In summary, about half of the UC patients with prior exposure to anti-TNF therapy showed a response to vedolizumab therapy. Furthermore, a report demonstrated that the drug persistency of second-line vedolizumab followed by anti-TNF therapy was significantly better than that of second-line anti-TNF followed by vedolizumab therapy in UC patients [32]. In contrast, there are limited data on the efficacy and safety of second-line ustekinumab or tofacitinib exclusively for UC patients with prior failure of anti-TNF therapy. Regarding no significant difference in effectiveness and drug persistency between ustekinumab, vedolizumab, and tofacitinib in our real-world data, vedolizumab could be selected as a comparable option in UC patients with prior failure of anti-TNF therapy.

There is a hypothesis that mucosal addressin cell adhesion molecule-1 (MAdCAM-1), a ligand of a4b7 integrin-positive T-cell, is consumed after anti-TNF therapy, reducing the effect of second-line vedolizumab blocking α4β7 integrin [33]. However, the serum MAdCAM-1 concentration did not decrease in non-responders to anti-TNF therapy [34]. In addition, it is still unknown whether the baseline concentration of serum MAdCAM-1 affects the efficacy of vedolizumab therapy. Therefore, it is thought premature to consider this hypothesis in clinical decision making at this time.

The reason for the failure of anti-TNF therapy was not found to be a significant factor associated with drug discontinuation of second-line biologics. Similarly, a previous report observed that refractoriness to anti-TNF therapy lowered the clinical response to followed vedolizumab but had no significant association with the vedolizumab persistency [35]. Clinical response and endoscopic improvement were identified as factors related to a low risk for discontinuing second-line biological therapy, and it is a consistent finding that endoscopic outcome affects drug persistency [30].

Our study has several limitations. Selection bias may exist due to the retrospective design and small sample size, although we made efforts to collect data from five institutions. Additionally, the efficacy evaluation did not include biochemical responses such as fecal calprotectin due to test implementation and timing variability. Since the most recently released ustekinumab had a relatively short follow-up period, further studies are needed to compare drug persistence.

In conclusion, ustekinumab, vedolizumab, and tofacitinib as second-line biological therapies showed comparable efficacy and drug persistency in UC patients with prior failure of anti-TNF therapy. A fine selection of second-line biologics is suitable rather than optimal sequencing.

## Figures and Tables

**Table 1:** Baseline characteristics of the study patients at second-line biological therapy.

	Ustekinumab (N = 11)	Vedolizumab (N = 40)	Tofacitinib (N = 19)	*p* Value
Age at diagnosis ^†^	36.0 ± 15.9	37.8 ± 17.2	36.5 ± 14.5	0.928 ^1^
Age at 2nd-line biological therapy ^†^	43.8 ± 21.5	43.6 ± 16.8	41.1 ± 13.5	0.856 ^1^
Disease duration, years ^†^	7.8 ± 7.9	5.8 ± 4.8	4.6 ± 3.2	0.875 ^2^
Sex				0.273 ^3^
Male	5 (45.5)	27 (67.5)	14 (73.7)	
Female	6 (54.5)	13 (32.5)	5 (26.3)	
Extent				0.725 ^3^
Left side	7 (63.6)	20 (50.0)	10 (52.6)	
Extensive	4 (36.4)	20 (50.0)	9 (47.4)	
Prior anti-TNF use				1.000 ^4^
1 ≥2	10 (90.9) 1 (9.1)	36 (90.0) 4 (10.0)	18 (94.7) 1 (5.3)	
Duration of anti-TNF, months ^†^	45.3 ± 40.4	19.5 ± 22.3	11.7 ± 10.8	0.077 ^2^
Discontinuation reason of anti-TNF				0.829 ^4^
Primary non-response	3 (27.3)	5 (12.5)	3 (15.8)	
Loss of response	7 (63.6)	29 (72.5)	14 (73.7)	
Adverse events	1 (9.1)	6 (15.0)	2 (10.5)	
Disease activity index				0.475 ^4^
Mayo score of 6–10	8 (72.7)	35 (87.5)	16 (84.2)	
Mayo score of 11–12	3 (27.3)	5 (12.5)	3 (15.8)	
Concomitant medication				
5-ASA	10 (90.9)	31 (77.5)	16 (84.2)	0.698 ^4^
Topical 5-ASA	2 (18.2)	4 (10.0)	7 (36.8)	0.041 ^4^
Steroid	7 (63.6)	17 (42.5)	8 (42.1)	0.430 ^3^
Immunomodulators	1 (9.1)	16 (40.0)	1 (5.3)	0.005 ^4^
Laboratory data				
Hemoglobin	12.6 ± 2.2	12.7 ± 2.1	12.6 ± 1.8	0.989 ^1^
CRP	0.9 ± 1.4	1.4 ± 1.9	0.9 ± 1.0	0.466 ^2^
Albumin	4.2 ± 0.5	4.0 ± 0.6	4.2 ± 0.5	0.437 ^2^
Follow-up duration, months ^†^	16.1 ± 4.9	34.0 ± 14.4	31.3 ± 7.2	<0.001 ^1^

*p* value was analyzed using ^1^ one-way analysis of variance (ANOVA), ^2^ Kruskal–Wallis test; ^3^ chi-squared test, and ^4^ Fisher’s exact test, respectively. Values are expressed as N (%) unless otherwise specified. ^†^ Mean ± standard deviation presented for continuous variables. 5-ASA, 5-aminosalicylic acid; CRP, C-reactive protein; anti-TNF, anti-tumor necrosis factor.

**Table 2:** Discontinuation reason and adverse events of 2nd-line biological therapies.

	Ustekinumab (N = 11)	Vedolizumab (N = 40)	Tofacitinib (N = 19)
Total number of discontinuations	0 (0.0)	14 (35.0)	4 (21.1)
Primary non-response	0 (0.0)	2 (5.0)	1 (5.3)
Loss of response	0 (0.0)	12 (30.0)	2 (10.5)
Adverse events	0(0.0)	0 (0.0)	3 (15.8)
With discontinuations	0 (0.0)	0 (0.0)	1 (5.3)
Without discontinuations	0 (0.0)	0 (0.0)	2 (10.5)

Values are expressed as N (%) unless otherwise specified.

**Table 3:** Associating factors with discontinuation of second-line biological therapies.

	Univariate		Multivariate		
	HR	*p* Value	HR	95% CI	*p* Value
Age at diagnosis	1.00	0.887			
Age at 2nd-line biological therapy	1.00	0.809			
Disease duration, years	0.93	0.222			
Male */female	0.66	0.438			
Left side */extensive	1.35	0.529			
Prior anti-TNF number of 1 */≥2	0.52	0.526			
Duration of anti-TNF, months	0.99	0.298			
Discontinuation reason of anti-TNF Primary non-response *					
Loss of response	1.57	0.554			
Adverse events	1.19	0.865			
Mayo score of 6–10 */11–12	0.68	0.605			
5-ASA No */Yes	0.55	0.257			
Topical 5-ASA No */Yes	1.36	0.591			
Steroid No */Yes	1.87	0.195			
Immunomodulators No */Yes	1.01	0.983			
Hemoglobin	0.93	0.522			
CRP	1.05	0.709			
Albumin	0.81	0.593			
Clinical response No */Yes	0.11	<0.001	0.09	0.03–0.31	<0.001
Endoscopic improvement No */Yes	0.17	<0.001	0.15	0.06–0.41	<0.001

* Reference. 5-ASA, 5-aminosalicylic acid; CRP, C-reactive protein; anti-TNF, anti-tumor necrosis factor.

## Data Availability

Data are available from the corresponding author upon reasonable request.
